# Regenerative medicine approaches for the treatment of peripheral nerve injuries: progress and challenges

**DOI:** 10.1093/rb/rbag015

**Published:** 2026-02-05

**Authors:** Haochen Yang, Qinwen Bao, Xiaosong Gu, Meng Cong

**Affiliations:** School of Medicine, Nantong University, Nantong 226001, China; School of Medicine, Nantong University, Nantong 226001, China; Key Laboratory of Neuroregeneration of Jiangsu and Ministry of Education, Co-Innovation Center of Neuroregeneration, Nantong University, Nantong 226001, China; Key Laboratory of Neuroregeneration of Jiangsu and Ministry of Education, Co-Innovation Center of Neuroregeneration, Nantong University, Nantong 226001, China

**Keywords:** peripheral nerve injury, regenerative medicine, cell and exosomes therapy, tissue engineering, gene therapy

## Abstract

Peripheral nerve injury (PNI), a prevalent clinical disorder induced by trauma, immune diseases and genetic factors, can result in sensory, motor and autonomic dysfunction, with this dysfunction seriously compromising patients’ quality of life. Although traditional treatment methods such as autologous nerve transplantation are the gold standard, there are limitations such as insufficient donors, poor repair effect of long segmental defects and low functional recovery rate. Effective repair after nerve injury is still a challenge in neurosurgery. Therefore, new strategies need to be found to treat peripheral nerve injuries. Regenerative medicine has attracted much attention as an effective alternative therapy to promote the repair and regeneration of damaged peripheral nerves. Regenerative medicine provides new ideas for breaking through the bottleneck of traditional treatment by integrating cutting-edge technologies such as cell therapy, tissue engineering and gene therapy. However, current regenerative medicine needs to overcome challenges such as efficacy stability, long-term safety and cost-effectiveness. In this review, we summarize the structure and function of peripheral nerves, the mechanism and classification of injury and the pathological progression of PNI. Importantly, regenerative medicine strategies for the treatment of PNI are emphasized, and the challenges and future development of regenerative medicine are envisioned.

## Graphical abstract

**Figure:**
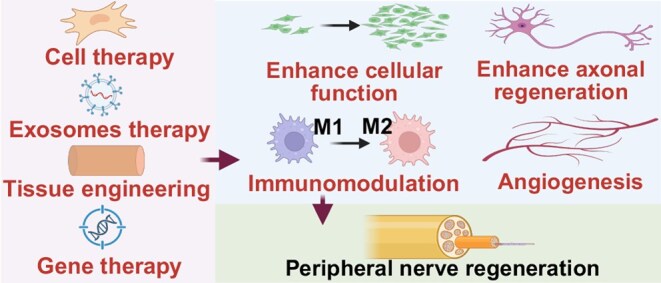

## Introduction

Peripheral nerve injury (PNI) is a common clinical problem with a high incidence worldwide, ranging from 13 to 23/100 000 person-years [1]. It can be caused by trauma (strain, cut and crush), disease (diabetic neuropathy and neuritis), chemical damage and congenital factors. PNI often results in reduced or complete loss of sensory function in damaged innervation areas, decreased or complete paralysis of damaged innervation muscles and autonomic dysfunction, reducing the quality of life of patients and bringing a heavy disease burden to patients and society [2].

The traditional treatment methods for peripheral nervous system (PNS) injury mainly include end-to-end anastomosis, autologous nerve transplantation and allogeneic nerve transplantation, among which autologous nerve transplantation is the gold standard treatment method for PNI [3]. However, these methods have many shortcomings, such as difficulty in achieving good repair effect for long-segment (>10 mm) nerve damage, insufficient donor nerves, high rate of lesions at donor sites and mismatch between donor nerves and recipient sites [4]. The findings revealed that less than 50% of patients who underwent peripheral nerve transplant surgery achieved sensory and motor recovery [5]. Besides surgery, medication is also an important treatment option. Neurotrophic factors such as nerve growth factor (NGF), brain-derived neurotrophic factor (BDNF) and fibroblast growth factor 2 have been widely studied and applied, which can promote the regeneration of axons and the formation of new myelin sheaths [6]. For example, fibroblast growth factor 2-transfected Schwann cells transplanted into the sciatic nerve defect in rats can effectively support the regeneration of myelin sheath fibers [7]. However, significant clinical challenges in their delivery remain, such as short drug half-life, difficulty in delivering accurately to the site of injury and blood-nerve barrier restrictions on effective uptake, which limit their effectiveness in clinical applications [6]. Therefore, the treatment of PNI remains a serious challenge.

In recent years, in response to these challenges, the emergence of regenerative medicine has brought new hope for the repair of PNI. Regenerative medicine is a field of medicine that seeks to repair or replace damaged tissues and organs. The core idea is to use biological principles and techniques to restore biological function by repairing, regenerating or replacing damaged cells, tissues or organs. It offers a range of innovative strategies and technologies, including cell therapies, biomaterials, gene therapy and growth factors, to promote the repair of damaged nerves or tissues and restore their function [8]. The advantage of regenerative medicine lies in its ability to accelerate nerve regeneration through exogenous intervention, improving the efficiency and quality of repair. For example, stem cell therapy can effectively promote nerve repair by directly guiding cells at the site of injury to differentiate into nerve cells or neural support cells [9]. In addition, biomaterials and scaffold technologies provide structural support for the repair of nerve damage, while growth factors and gene therapies enhance the ability of nerve regeneration by modulating the local microenvironment [10]. Relying on regenerative medicine technology, scientists have made some breakthroughs, especially in the early intervention after nerve injury and the treatment of long-term nerve defects. However, despite the promising application of regenerative medicine in neural repair, it still faces challenges in terms of efficacy, ethics and cost.

In this study, we introduce a comprehensive review of the advancements in the repair of peripheral nerve injuries, with an emphasis on the potential of tissue engineering and regenerative medicine approaches for PNI, including cell transplantation, tissue-engineered construct implantation and other potential therapeutic strategies. It integrates insights from neuroscience, regenerative medicine and materials science, providing a multifaceted perspective for the repair of PNI. By examining the events of regeneration after PNI and evaluating existing strategies, this study showed the advantages and limitations of current approaches, such as donor site morbidity and immune rejection issues. This research includes a detailed exploration of the progress in different strategies and further discussion of potential future research directions. This study proposes potential avenues for future research, aiming to encourage researchers to explore innovative approaches in regenerative medicine-based nerve repair. By integrating insights from multiple disciplines, this work contributes to the advancement of the frontiers of PNI treatment.

## Pathophysiology of PNI

### The structure and function of peripheral nerve

The PNS is an important part of the nervous system, composed of nerve cells, supportive cells and nerve fibers. Nerve cells are basic cells found in PNS, with dendrites, cell bodies and axons, among which axons are responsible for transmitting information to distant effector organs or receiving information from the outside world [11]. Supporting cells in PNS mainly include Schwann cells, macrophages and satellite glial cells, which play an important role in axon regeneration [12–14]. Schwann cells can produce extracellular matrix (ECM), cell adhesion molecules, integrins and neurotrophic factors, which provide the appropriate microenvironment for cells in the tissue to promote axon regeneration of damaged nerves [15]. Macrophages play important roles in angiogenesis, migration of Schwann cells, clearance of myelin and axon debris and growth of regenerative neurons [16]. Satellite glial cells can secrete ECM components and NGF, forming a suitable microenvironment for axon growth, guiding axon growth along a specific path and promoting the reconstruction of neural connections [17]. Nerve fibers are composed of axons and their surrounding myelin sheaths, which are formed by lipid substances secreted by Schwann cells and have an electrical insulating effect, which significantly increases the speed of nerve signaling [18]. The function of peripheral nerves mainly includes sensory, motor and autonomic functions and these function depends on their complex tissue structure and physiological characteristics [19]. Axons complete sensory, motor and regulatory functions through electrochemical signals and neurotransmitters are released in the synaptic gap, further amplifying the signal [20]. Nerve fiber damage can lead to signal interruption, resulting in sensory impairment, muscle weakness or autonomic dysfunction. The repair of damage depends on the regeneration ability of nerve cells, the supporting role of Schwann cells, the remodeling of the cytoskeleton and the regulation of inflammatory responses [21].

### Mechanism and classification of PNI

The mechanism of PNI is complex, usually caused by mechanical, chemical or ischemic factors. Different injury types trigger different pathological reactions and the severity of injury, location and treatment methods jointly affect the difficulty and effectiveness of nerve repair [22]. Among them, mechanical injuries are the most common, covering three forms: compression, pulling and cutting. Compressive injury is mostly caused by external pressure. In the early injury stage, nerve conduction is only temporarily blocked; whereas persistent compression can induce nerve fiber fracture and myelin sheath degeneration. Traction injury arises from external pulling forces, often accompanied by axon laceration—in severe cases, nerve fibers rupture completely, posing great challenges to repair. Cutting injury is characterized by nerve rupture or transection, which usually requires surgical intervention; however, axon regeneration is constrained by the condition of the injury site and the local regenerative microenvironment [23–25]. As for chemical injury, it is frequently induced by toxic substances or metabolic disorders. For instance, chemotherapeutic drugs and antibiotics can directly act on Schwann cells or nerve fibers, thereby causing myelin degeneration and subsequent loss of nerve function. For example, diabetic neuropathy causes hyperglycemia to damage blood vessels and affect the supply of nerve nutrients. Such injuries gradually appear after long-term exposure to harmful substances, with clinical manifestations such as numbness, pain, muscle weakness and other neurological functions gradually declining [26, 27]. Ischemic injury is caused by a lack of blood flow and usually occurs in the case of external compression or vascular disease. Due to the high demand for oxygen and nutrients in neural tissue, ischemia can rapidly lead to cellular hypoxia and energy metabolism disorders, inducing apoptosis and inflammation. Increased intracellular pressure and acidosis occur in nerve cells and Schwann cells, which eventually lead to irreversible damage, slow repair process and poor effect, especially when the duration of ischemia is too long [28, 29]. In addition, nerve damage caused by immune response has gradually attracted attention, such as idiopathic neuritis and post-infection syndrome, which can lead to peripheral nerve damage. Abnormal immune system attacks peripheral nerves, inducing inflammation and dysfunction [30]. Therefore, the treatment of PNI should be personalized according to the specific type of injury and the implementation of intervention. Mechanical injuries are primarily repaired by surgery; chemical injuries are treated by adjusting drug use and regulating metabolism. For ischemic injury, the emphasis is on early restoration of blood supply and protection against oxidative stress to avoid further nerve damage [27, 29].

**Figure 1:**
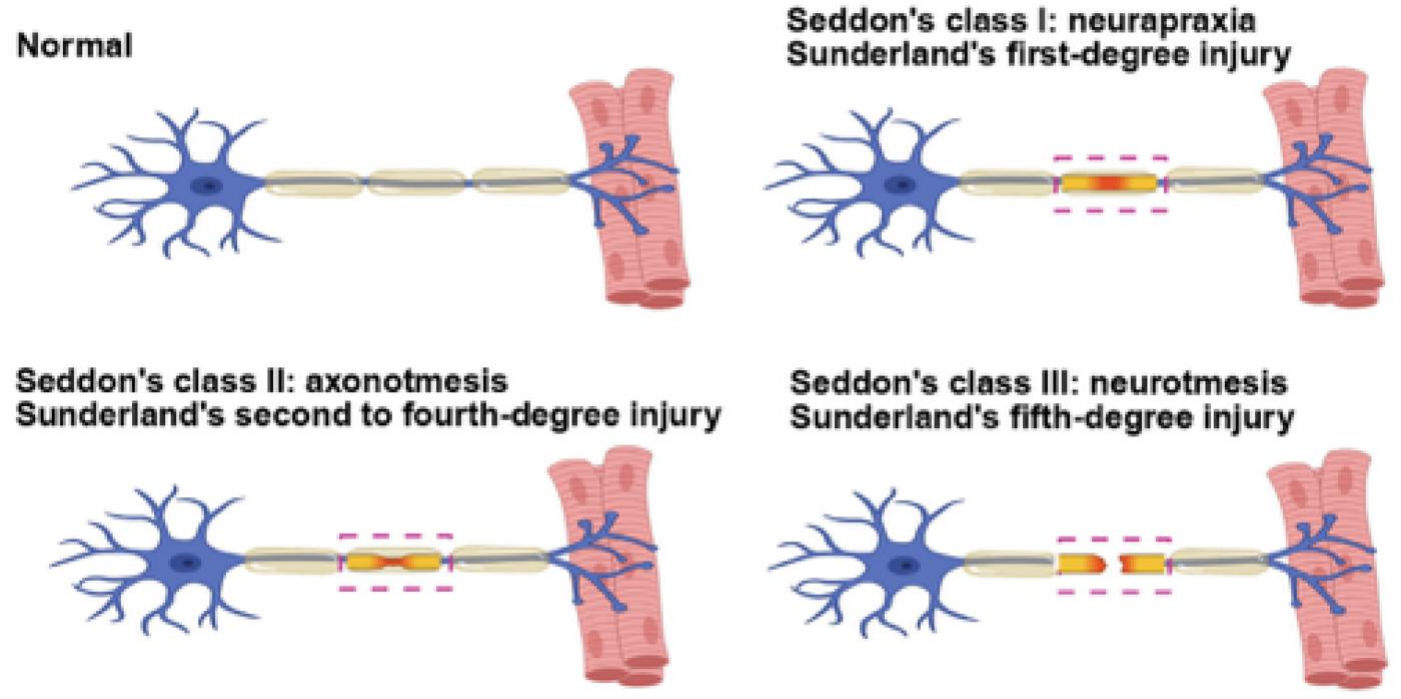
Schematic representation of the classification of PNI. The severity of PNI is classified as class I (neurapraxia), class II (axonotmesis) or class III (neurotmesis) by Seddon and first- to fifth-degree by Sunderland.

Peripheral nerve injuries are mainly classified by Seddon [31] and Sunderland (Figure 1 and Table 1) [34]. The Seddon protocol [31] classifies PNI as neuropraxia, axonotmesis and neurotmesis. Neuropraxia is a local demyelination at the site of injury without axon and peripheral connective tissue degeneration, and is the mildest form of PNI. However, the recovery process for this type of injury varies widely, and full recovery is usually achieved within days or up to months [35]. The characteristic of axonotmesis is the cleavage of the axon and its myelin sheath, which can lead to Waller’s degeneration, but the endoneurium and bundle membrane may be destroyed. The prognosis of axonotmesis mainly depends on the distance between the injury site and the target organ and the self-regeneration ability is limited, generally requiring appropriate surgical intervention [32]. Neurotmesis refers to complete damage to the peripheral nerves and is the most serious type of PNI. In neurotmesis, the endoneurium, perineurium and epineurium are all completely severed, which results in ruptures of axons, myelin sheaths and connective tissue. The prognosis for neurotmesis is poor, and recovery without surgery or other treatment options is almost non-existent [33]. The Sunderland classification [34] classifies PNIs into five categories based on increased severity of nerve damage [37, 38]. Grade I refers to focal segmental demyelination without axon injury, corresponding to neuropraxia in the Seddon classification; Grade II refers to the damage of the axon, but the endoneurium is intact. If the damaged site is close to the target organ, it may be close to normal recovery; Grade III means that the axon and endoneurium are damaged, but there is an intact perineurium, and the continuity of the tract is preserved; Grade IV means that the axon, endoneurium and perineurium are damaged and only the epineurium remains intact; Grade V refers to a complete defect of nerves, complete destruction of axons and surrounding connective tissue, which corresponds to neurotmesis in the Seddon classification. In addition, most PNIs may exhibit a combination of multiple injury types, referred to as mixed injury patterns [39].

**Table 1:** Seddon and Sunderland classification of PNI.

Seddon classification	Sunderland classification	Severity	Reasons of injury	Damage characteristics	Spontaneous recovery		Nerve conduction research	Electromyography (EMG)	References
Neuropraxia	Grade I	The mildest class of PNI.	Ischemia, traction or mild compression	No axon injury, focal segmental demyelination	Yes, in days or up to 3 months.		Proximal partial or complete conduction block, distal conduction function is usually preserved after several weeks.	Muscle morphology was normal and poor motor unit action potential (MUAP) recruitment.	[31–33]
Axonotmesis	Grade II	More severe than neuropraxia.	Stretch, crush, percussion, electric shock or burn	The axon is damaged, the endoneurium is intact	The prognosis depends on the distance between the injury site and the target organ and the ability to self-regenerate is limited	Yes, slower than neuropraxia	Partial or complete block, with Wallerian degeneration at the distal end of injury 24-36 after PNI.	Abnormal activity	[34, 35]
	Grade III			Damaged axon and endoneurium with perineurium intact		Unlikely, surgical intervention may be needed.	Proximal block, while distal may still have some conduction function before Wallerian degeneration.		[35]
	Grade IV			Axon, endoneurium and perineurium are damaged, the epineurium is intact		Extremely unlikely, surgical intervention is required.	Both proximal and distal conduction blocks.		[22, 36]
Neurotmesis	Grade V	The most severe degree of PNI.	Sharp instrument injury, severe traction injury or injection of neurotoxic drugs	Complete nerve transection, axon, myelin sheath, endocardium, perineurium and epineurium were all broken.	No, surgical intervention is required		Complete conduction block proximally and distally.	Abnormal activity	[37]

### Pathological progress of PNI

Recent studies have shown that regeneration disorders following PNI are caused by the synergistic action of multiple factors, rather than simply due to difficulties in axon growth. Current research focuses on the following aspects: first, the inflammatory response, which can be divided into acute and chronic phases. In the acute phase, inflammation helps repair peripheral nerves and engulf damaged cells, thus, achieving a protective effect, while in the chronic phase, excessive inflammation may lead to the deterioration of injured nerve cells [40]. After PNI occurs, Schwann cells, macrophages and glial cells in the damaged area initiate an acute inflammatory response, which helps to clear the dead tissue and activate repair mechanisms. Macrophages are a “double-edged sword” in PNI repair. On the one hand, they effectively promote axon and myelin regeneration by removing residual myelin debris, secreting growth-promoting factors and inducing angiogenesis. On the other hand, they release inflammatory mediators, and an excessive inflammatory response can lead to the degeneration and necrosis of tissue cells, aggravating the degree of damage and inhibiting nerve regeneration [41, 42]. Studies have shown that following nerve injury, inflammatory factors, such as tumor necrosis factor alpha (TNF-α) and interleukin (IL) -1β, are significantly increased in concentration in the damaged area, and these factors play an important role in regulating the survival and regeneration of nerve cells [43]. In addition, regulation of the inflammatory response involves not only the activation of immune cells but also the interaction between nerve cells and Schwann cells. During the initial stages of the inflammatory response, Schwann cells are able to promote inflammation, and as the nerve repair process advances, their function shifts to support nerve regeneration [44, 45]. This inflammatory imbalance presents a key therapeutic target. Mesenchymal stem cell (MSC) therapy and particularly MSC-derived exosomes (EXOs) have been shown to modulate this response by promoting the polarization of macrophages from a pro-inflammatory M1 to a pro-regenerative M2 phenotype, and by secreting anti-inflammatory factors such as IL-10 and TNF-Ra, thereby creating a more favorable microenvironment for regeneration [46, 47].

Second, neurodegeneration, as an important pathological change, will cause rupture, degeneration and demyelination after axon injury. In severe cases, nerve cells develop distal degeneration, also known as Wallerian degeneration. To promote nerve regeneration, Schwann cells reactivate genes that promote nerve regeneration through a process of “dedifferentiation.” Such dedifferentiated Schwann cells are often referred to as activated Schwann cells [48]. When activated, Schwann cells secrete molecules such as inflammatory mediators and neurotrophic factors, attracting inflammatory cells and blood cells to the site of injury and removing myelin debris [49, 50]. Subsequently, Schwann cells enter a state of proliferation and are arranged in Bungner bands, providing a scaffold for axon regeneration, which can promote the extension of axons to target organs, thereby restoring the physiological function of nerves [51]. In addition, fibroblasts around the injury site proliferate after activation, and the cells promote axon regeneration by secreting neurotrophic factors (such as BDNF). If excessive proliferation, scar tissue will be formed to hinder axon regeneration [52, 53]. The physical and chemical barrier posed by scar tissue is a major obstacle addressed by tissue engineering strategies. Biomaterial scaffolds, especially those made from natural polymers like chitosan or alginate, are designed to provide a permissive physical pathway that bridges the nerve gap, directing axon growth away from inhibitory scar regions. Furthermore, these scaffolds can be functionalized with enzymes (e.g. chondroitinase ABC) to actively degrade inhibitory chondroitin sulfate proteoglycans [54–56]. Following axon transection, the proximal portion undergoes retrograde degeneration extending to the adjacent node of Ranvier, which subsequently form a growth cone. Growth cones are structures rich in actin and microtubules that exhibit high motility, capable of sensing both chemical signals (such as neurotrophic factors) and physical signals (such as the hardness and porosity of the ECM in their surroundings, guiding axons to extend in a direction conducive to their growth [57, 58]). However, the rate of axon regeneration is relatively slow, approximately 1–2 mm per day. Its growth depends on various factors, such as the appropriate ECM, sufficient neurotrophic factors and guided channels formed by Schwann cells [59]. Changes in inflammatory factors, cytokines and ECM in the microenvironment may inhibit axon growth or lead to fibrosis [60]. Growth cones guide regeneration through cytoskeleton remodeling, adhesion molecules and ECM interactions. The guidance of growth cones is influenced by chemical signals and mechanical resistance, and misaligned guidance may cause axons to vagrant and fail to accurately reach the target tissue [61, 62].

Finally, there is the mechanism of aplastic dysfunction. Although peripheral nerves can repair themselves to some extent in mild injuries, in severe or complex injuries, nerve regeneration is often affected by a variety of disorders. The current study found that changes in the microenvironment at the site of injury are one of the key factors in neural regeneration [63]. First, nerve scars formed after injury are a major obstacle to the repair of peripheral nerve injuries and consist of glial scars formed by the proliferation of fibroblasts and glial cells [64]. Scar tissue forms a physical barrier near the broken end, secreting inhibitory molecules such as chondroitin sulfate proteoglycans, which prevent axons from passing through [65]. Removing or reducing scar tissue is one of the important strategies for improving repair outcomes. Second, cytokines generated by the inflammatory response and oxidative stress can inhibit the activity of axon growth factor and NGF, reducing the efficiency of nerve repair [54, 66]. In addition, the function of Schwann cells is crucial during neural regeneration. Schwann cells not only provide physical support during the regeneration of axons but also promote nerve growth by secreting growth factors. As the damage worsens and the repair process is delayed, Schwann cells may gradually lose their function, leading to a decrease in regeneration efficiency [67]. Given the pivotal role of Schwann cells, a primary goal of regenerative medicine is to support and enhance their function. Cell therapy directly addresses this by transplanting exogenous Schwann cells or stem cell-derived Schwann-like cells to repopulate the injury site. Moreover, EXOs therapy has emerged as a powerful strategy, with evidence showing that EXOs from various sources can be internalized by endogenous Schwann cells to promote their proliferation, migration and secretion of neurotrophic factors, effectively rescuing their repair phenotype [68–70].

**Figure 2:**
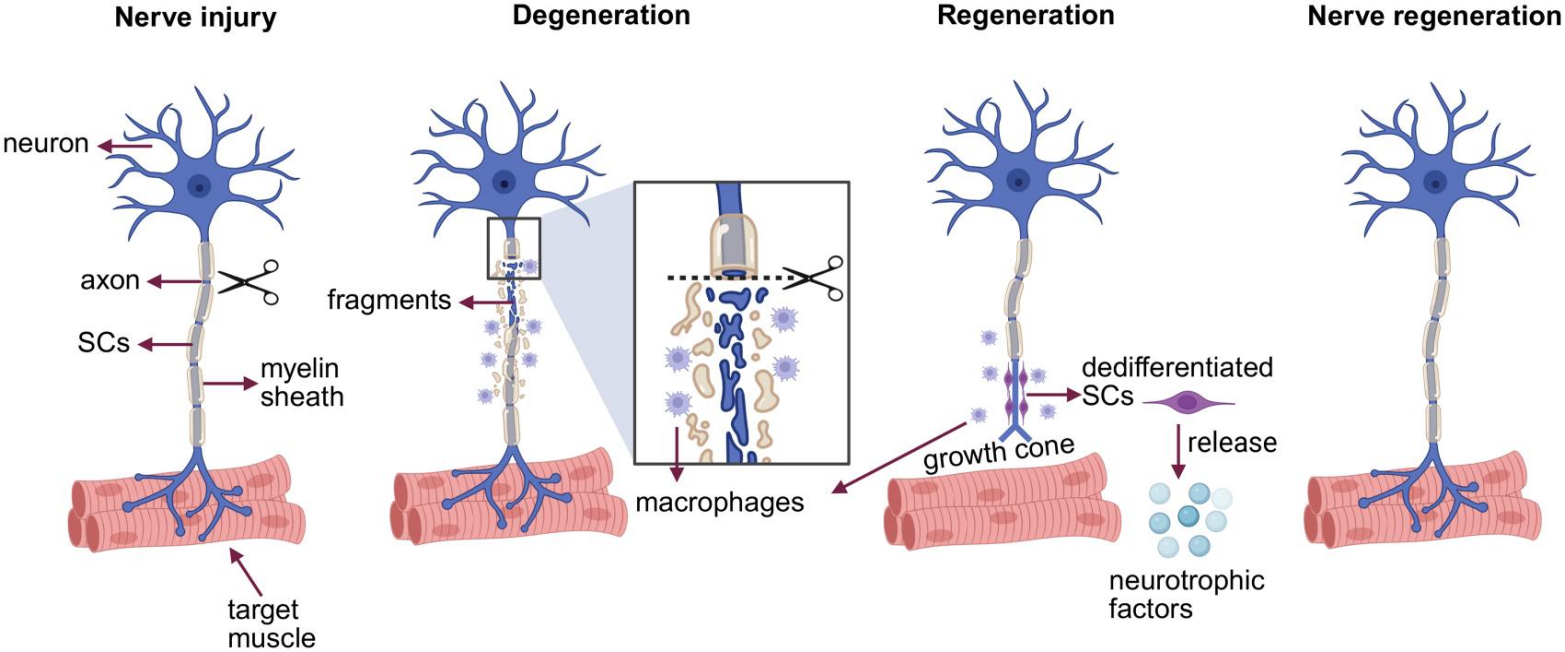
The schematic illustration of the events after PNI and regeneration. Following injury, the distal neuron undergoes a process known as Wallerian degeneration. After injury, the axon ruptures, leading to degeneration and fragmentation of the myelin sheath. Schwann cells rapidly proliferate, and the proliferating and recruited macrophages phagocytize and clear the fragments of axon and myelin. When the fragments are removed, the related cytokines from Schwann cells are upregulated, forming a favorable environment and axonal regeneration.

In conclusion, the pathophysiological process of PNI is a complex process intertwined by multiple factors (Figure 2). Inflammation, neurodegeneration and aplasia are the key problems to be solved in the process of nerve injury repair. Current research continues to shed light on the cellular and molecular mechanisms involved in this process, providing an important theoretical foundation for the development of therapeutic strategies. However, to achieve complete neural repair and functional recovery, greater breakthroughs are still needed in the fields of cell therapy and biomaterials.

## Regenerative medicine therapeutic approaches

### Cell therapy

**Figure 3:**
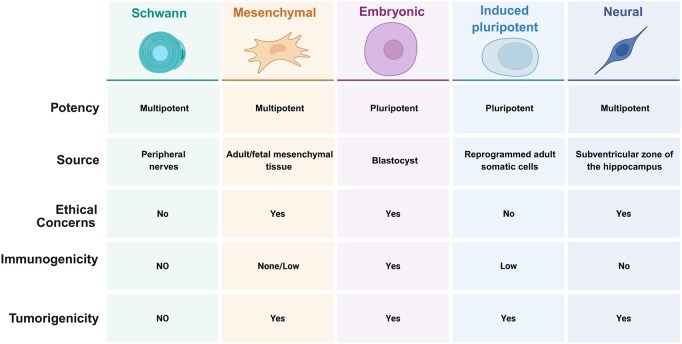
Properties of commonly used cell types for PNI treatment. All cell transplantations are assumed to be autologous except for that of neural progenitors and embryonic stem cells. The columns represent the specific cell type used, and the rows represent specific characteristics associated with their use.

As an important therapeutic strategy in regenerative medicine, cell therapy has shown wide application potential in the repair of PNI (Figure 3) [71]. The repair process after nerve injury is very complex, involving the proliferation, differentiation, migration of nerve cells and the reconstruction of the injury site microenvironment [72]. Cell therapy promotes the repair and regeneration of damaged nerves by transplanting exogenous cells or regulating the function of endogenous cells. The main research objects of exogenous cell therapy include Schwann cells, stem cells and glial cells. These cells can not only directly replenish missing or damaged nerve cells but also become an important means of nerve repair by secreting neurotrophic factors, modulating the local inflammatory environment and guiding axon extension [73, 74]. Activation of endogenous cells promotes the improvement of the microenvironment by stimulating the function of Schwann cells or immune cells at the site of injury, thus, promoting neural regeneration [75]. Furthermore, studies have confirmed paracrine roles played by various types of cells in tissue protection and repair, and these beneficial effects may be mediated primarily or in part by the release of soluble factors, such as EXOs released by cells to injection sites (*in vivo*) or conditioned media (*in vitro*) [76, 77].

#### Schwann cells therapy

Schwann cells are the most abundant glial cells in the PNS and play an important role in nerve regeneration. After PNI, Schwann cells can promote the growth and recovery of nerve cells, remove debris from the damaged area, form myelin sheaths and promote axon regeneration [15]. The transplantation of Schwann cells is considered an ideal therapeutic strategy, especially for a wide range of peripheral nerve injuries. Schwann cells promote the regeneration of damaged nerves by secreting ECM components and neurotrophic factors, such as NGF, BDNF and neurotrophic factor-3 (NT-3) [78]. Studies have shown that Schwann cell transplantation combined with neural scaffolds can effectively promote the repair of damaged peripheral nerves [79]. Sun et al. co-cultured Schwann cells with allogeneic acellular nerves *in vitro*, and the experimental results showed that acellular nerves inoculated with Schwann cells could enhance nerve regeneration and functional recovery after bridging the sciatic nerve space in rats [80]. Huang et al. collected EXOs of endothelial cells (ECs) and demonstrated that ECs-derived EXOs (ECs-EXOs) had good affinity for nerve cells *in vitro* and *in vivo*. Their further studies revealed that ECs-EXOs could promote and maintain the repair-related phenotype of Schwann cells to enhance axon regeneration, myelin regeneration and angiogenesis, thereby promoting functional recovery after PNI [68]. However, Schwann cells have a relatively limited source and are susceptible to losing their function when cultured *in vitro*. In addition, the transplantation of Schwann cells may induce immune rejection. Although this rejection response is less pronounced than that associated with other cell types, the long-term effects and safety profile of Schwann cell transplantation require further assessment [81].

#### Stem cells therapy

Stem cells are a kind of special cells with self-renewal ability and multi-directional differentiation potential, which are widely used in the field of regenerative medicine [82]. During the repair of PNI, stem cells can differentiate into nerve cells, Schwann cells and other supportive cells, which can promote nerve regeneration and repair at the injured site. According to their different sources, stem cells can be divided into embryonic stem cells (ESCs), induced pluripotent stem cells (iPSCs) and adult stem cells. Adult stem cells are a type of cells derived from postnatal tissues with multipotent differentiation potential, including MSCs and neural stem cells (NSCs). Each of these cell types has distinct characteristics and application prospects [83]. Transplanted stem cells play a crucial role in promoting neural regeneration through a variety of mechanisms, such as stem cell differentiation and neural regeneration, cytokine secretion and regulation and regulation of the immune microenvironment [84]. From the perspective of differentiation and neural regeneration, stem cells initiate their differentiation mechanism after receiving damage signals and gradually transform into cell types with specific functions. MSCs can differentiate into Schwann-like cells, which are responsible for forming myelin sheaths and are essential for repairing damaged axons and restoring nerve conduction function [85]. IPSCs can differentiate into nerve cell-like cells under stimulation to replace damaged nerve cells, helping to build neural networks and improve neural function [86]. After nerve damage, factors such as local inflammation and necrosis provide induction signals for stem cell differentiation. Stem cells secrete signaling molecules such as transforming growth factor-β (TGF-β) and epidermal growth factor (EGF) to activate related pathways, differentiate into functional cells, replenish cells in damaged areas, regulate axon growth direction and rebuild neural networks. What’s more, after differentiation, stem cells form synapses through axon extension, establish stable connections with host cells and transmit information, thus, providing a structural and functional basis for neural repair [87, 88]. In terms of the secretion and regulation of cytokines, neurotrophic factors such as BDNF, NGF and glial cell-derived neurotrophic factor (GDNF) secreted by stem cells promote the survival of nerve cells and activate axon growth signaling pathways, thereby accelerating the process of nerve regeneration. In addition, stem cells also reduce the damage to surrounding tissues by secreting anti-inflammatory factors such as IL-10 and tumor necrosis factor receptor antagonist (TNF-Ra). They secrete matrix metalloproteinase (MMP) to regulate the ECM in the damaged area, which promotes axon extension and nerve fiber rearrangement. Moreover, they stimulate angiogenesis by modulating angiogenic factors such as endothelial growth factor (VEGF) and platelet-derived growth factor (PDGF) [71, 89]. In terms of immune microenvironment regulation, stem cells induce the phenotypic transformation of macrophages by secreting anti-inflammatory and immunosuppressive factors; direct contact with immune cells through expression of programmed death ligand-1 (PD-L1) inhibits immune rejection and reduces the risk of immune rejection induced by allogeneic transplantation [90].

MSCs are a class of mesoderm-derived pluripotent stem cells with high self-renewal ability, pluripotent differentiation potential and low immunogenicity, and can maintain their biological characteristics after expansion *in vitro* [91, 92]. MSCs come from a wide range of sources, including bone marrow (bone marrow-derived MSCs [BMSCs]), adipose tissue (adipose-derived MSCs [ADMSCs]), umbilical cord (umbilical cord-derived MSCs [UMSCs]), skin (skin-derived MSCs [SKMSCs]) and placenta (placental-derived MSCs [PMSCs]) [93]. Under the influence of different induction media *in vitro*, MSCs can differentiate into chondrocytes, osteoblasts, adipocytes, nerve cells and glial cells [94]. For example, Wang et al. [95] showed that BMSCs were able to differentiate into the Schwann cell phenotype *in vitro* after co-culturing with sciatic nerve extracts from degenerative rats. ADMSCs can transdifferentiate into Schwann-like cells to enhance neural regeneration [96]. In addition, MSCs can secrete a variety of growth factors through paracrine action, such as BDNF, NGF and VEGF, which play a significant role in the regulation of axon regeneration, angiogenesis and inflammatory response and promote nerve regeneration. For example, Dezawa et al. [97] used retroviral vectors to obtain GFP-expressing BMSCs (GFP-BMSCs) and subsequently anastomosed artificial grafts to the tangential end of the rat sciatic nerve. After 3 weeks, a large number of newly formed fibers were observed, and BMSCs were found to have myelination, indicating that BMSCs can differentiate nerve cell-like cells and secrete a large number of NGF to induce axon growth. Studies have shown that after systemic injection of adipose-derived stem cells (ADSCs), some cells can migrate to the site of nerve injury, help reduce inflammation and release NGF such as insulin-like growth factor I and BDNF to promote nerve regeneration [98]. The study also revealed that ADSCs play an immunomodulatory role by prompting host Schwann cells to increase the production of BDNF and GDNF [99]. However, despite the promising results of MSCs in preclinical studies, they still face challenges such as low survival rates of transplanted cells, limited efficacy and difficulty in maintaining long-term function after transplantation [100].

ESCs are pluripotent stem cells, which are obtained by *in vitro* fertilization technology and have significant proliferative capacity and differentiation potential. They can differentiate into three germ layers of embryos and form various types of cells or tissues except fetal cells [101]. Studies have shown that differentiating ESCs into nerve cells or Schwann cells and transplanting them to nerve injury sites can significantly promote axon growth and functional recovery. For example, in a mouse model of sciatic nerve injury, embryonic stem cell-derived neural progenitor cells (ESCs-NPCs) were transplanted to the injury site, and ESCs-NPCs were able to differentiate into Schwann-like cells to promote axon regeneration, myelin sheath regeneration and sciatic nerve function restoration [102]; neural crest cells (NCCs) differentiated from human ESCs can secrete a series of bioactive nutritional factors such as BDNF, NGF and angiogenesis factor, which can stimulate the growth of nerve processes when co-cultured with nerve cells *in vitro*. When the nutritional factors are incorporated into artificial nerve grafts, they can promote sciatic nerve regeneration in injured rats [103]. In addition, SCs derived from human ESCs can not only express Schwann cells markers such as transcription factors Slug (Snail2), S100A, Sox9 and p75 but also induce myelination in DRG nerve cells [104]. Studies have shown that after 1 cm of sciatic nerve transection in rats, NPCs derived from mouse ESCs were directly microinjected into the outer membrane of peripheral nerves as natural catheters, a process that promoted extensive restoration of morphology and function. The injected stem cells can survive for up to 3 months after cell transplantation and differentiate into myelin-forming cells [102]. However, ESCs may still undergo poor differentiation or excessive proliferation after transplantation, such as the development of teratomas, which increases the potential safety risks. Due to the allogeneic nature of the transplanted ESCs, the immune response is also an inherent consideration. In addition, the application of ESCs is subject to ethical and legal restrictions, limiting the possibilities for clinical use [101]. The development of iPSCs technology offers a new way to address this issue. iPSCs are obtained by reprogramming somatic cells, avoiding ethical controversy while having differentiation potential similar to ESCs [105].

In recent years, iPSCs have become one of the hotspots in the treatment of nerve injury. Studies have shown that by differentiating undifferentiated iPSCs into neural crest stem cells and Schwann cells with myelination ability, peripheral nerve function can be effectively restored [104]. In addition, its development has expanded cell sources for cell therapy and has largely contributed to advances in regenerative medicine [105]. Differentiation of human iPSCs into Schwann cells expressing SRY-Box transcription factor 10 (SOX10), S100b and glial fibrillary acidic protein (GFAP), which can express myelin markers (MBP, MPZ and glial protein), migrate and form myelin when co-cultured with iPSC-derived motor nerve cells, which helps repair PNI [106]. One study showed that iPSCs promoted axon regeneration and myelination at both 24 and 48 weeks postoperatively, without inducing teratomas [107]. Kim et al. successfully established a method for generating Schwann cell precursors (SCPs) from human pluripotent stem cells (hPSCs) and differentiating them into Schwann cells. The experimental results show that hPSCs-SCP-SCs can form myelin sheaths and secrete neurotrophic factors when co-cultured with embryonic rat dorsal root ganglion nerve cells *in vitro*, and transplanted into a mouse model of sciatic nerve injury can promote nerve fiber regeneration, integration into axons and improve sciatic nerve function [108]. It has been reported that neural crest-like cells can promote axon elongation, myelin sheath regeneration and enhance the recovery of neural function, producing similar effects to the autograft group [109]. For example, low-intensity pulsed ultrasound (LIPUS) can promote the proliferation and cell viability of iPSCs-derived neural crest stem cells (iPSCs-NCSC), and promote the differentiation of iPSCs-NCSC into nerve cells and Schwann cells, and improve the regeneration and functional recovery of sciatic nerves in rats with 1 cm injury [110]. However, there are still potential safety concerns with iPSCs, including immune rejection, the risk of tumor formation after transplantation and the uncertainty caused by gene editing. These problems limit its clinical application as a seed cell in tissue-engineered neural grafts, necessitating further technological optimization [111].

Other notable studies have made use of stem cells with the ability to self-renew and differentiate into multiple cell types [112]. NSCs are progenitor cells with pluripotency and self-renewal ability. As primitive cells in the nervous system, they can directly differentiate into nerve cells, glial cells and other nerve tissue cells. They have important applications in the treatment of central nervous system and peripheral nerve injuries [113]. NSCs transplantation can form new nerve cells and Schwann cells in the injured area of peripheral nerves, secrete many neurotrophic factors, such as BDNF, fibroblast growth factor, NGF and insulin-like growth factor, thereby promoting angiogenesis, nerve growth and myelination [114]. Lee et al. [115] showed that in a model of sciatic nerve injury, transplantation of NSCs into mice expressing VEGF enhanced fiber conduction and myelin regeneration. Studies have confirmed that interleukin-12p80 can induce NSCs to differentiate into myelin Schwann cells [116]. In addition, catheters implanted with NSCs and IL12p80 in a mouse model of sciatic nerve injury promoted nerve regeneration and improved motor function recovery [116]. However, the safety of neural stem cell transplantation and its long-term effectiveness remain controversial, especially in preventing excessive proliferation and tumor formation, which remains a key issue [117].

For long-gap PNI repair, different stem cell types show distinct *in vivo* outcomes: MSCs especially ADMSCs exhibit the most balanced performance, with high survival rates (60–70% at 8 weeks) and significant axon regeneration promotion (nerve conduction velocity recovery rate ∼50% in 15 mm defects) [118]. iPSCs show strong myelination ability (myelin sheath thickness ∼80% of normal nerves) but carry tumorigenicity risks [107]. NSCs have high differentiation specificity but poor survival in harsh injury microenvironments (survival rate < 30% in 20 mm defects) [114]. ESCs demonstrate excellent regeneration potential but are limited by ethical issues and immune rejection [102]. Overall, MSCs are the most clinically translatable for long-gap repair, while iPSCs hold promise with further risk mitigation.

#### Clinical translation of stem cell therapy

The clinical trial of PNI consists of three stages. The goal of the first phase of the trial is to evaluate the safety of treatment and potential harmful or toxic effects before performing cell transplantation on humans. In the second stage, the goal is to determine the potential and efficacy of the treatment compared to the control group. Usually, participants are recruited and randomly and double-blindly assigned to either the experimental group or the control group. Phase 3 clinical trials are usually the final clinical trials. The goal is to confirm the preliminary results obtained in Phase 2 by recruiting more participants, demonstrating that the therapeutic intervention has significant clinical advantages statistically. Some clinical trials, such as NCT02387749, NCT04346680 and NCT04870067, are investigating the benefits and limitations of stem cell-based therapies for patients with PNS diseases. The conduct of these studies helps to determine the efficacy of treating these diseases and identify potential limitations in clinical applications. Most of these studies are in the first or second stage. At the time of writing this review, registered clinical trials for stem cell therapy for PNI are listed on www.clinicaltrials.gov.

For example, with regard to diabetes peripheral neuropathy, 10 patients participated in the study of the benefits of BMSCs as potential treatments (NCT02387749). This study examined how a single intravenous injection of BMSCs improved symptoms such as pain, sensory loss and nerve conduction. The results showed that after 90 days of injection, blood glucose levels and neurological function improved, while bFGF and VEGF levels increased. In a nonrandomized, open-label study on the safety and efficacy of using undifferentiated autologous ADSCs, 10 injections of ADSCs were directly administered to the nerves. Patients who receive clinical and electrophysiological follow-up for ≥2 years will undergo clinical evaluation, electrophysiological evaluation and electromyography evaluation to assess safety and efficacy (NCT04346680). In addition to clinical trials, a recent case report described a 71-year-old patient using ADMSCs to treat superior gluteal nerve axonal injury. After conventional rehabilitation failed, MSC infiltration was chosen and performed with ultrasound guidance. Two months later, the patient showed normalization of electromyography, indicating full nerve recovery, along with significant improvement in neuropathic pain. The patient also demonstrated a 55% increase in maximum torque and a 9% increase in power during right hip extension in isokinetic evaluation, resulting in improvement of muscle strength and functionality [119]. Another phase 3 clinical study investigated the efficacy of WJMSCs in treating severe toxic optic neuropathy resistant to current therapies [120]. The study included 36 eyes from 18 patients, and treatment significantly improved visual outcomes (NCT04877067).

The limited clinical evidence reveals significant challenges in translating promising experimental results into human applications. The stark contrast between successful case reports [119] and negative case series [121] emphasizes the importance of multiple factors, including stem cell sources and preparation, delivery methods, patient selection, intervention timing and outcome evaluation. Successful clinical cases utilized autologous adipose-derived MSCs delivered through ultrasound-guided local injection, while unsuccessful series utilized heterologous cell sources with various delivery methods, indicating that autologous cells and precise delivery may be crucial for clinical success.

Despite the broad prospects, there are still some unsettling details that affect the safety and efficiency of cell therapy. For cell-based therapies, the applied cells should be collected and cultured in advance, expanded to a large population and frozen before transplantation. Many attempts can be made to advance the clinical application of cells. For example, attention should be paid to the tumorigenicity of cells. Cell banks can be constructed and preserved to ensure the quantity and quality of cells. Cells can be preprocessed through gene editing. The mobilization, homing, migration and delivery methods of cells should be further improved to enhance the survival rate of the applied cells.

### Cell-derived exosomes therapy

Studies have shown that EXOs are extracellular vesicles (EVs) carrying diverse cargo [122–124]. EVs can be divided into apoptotic bodies, micro-vesicles (MVs) and EXOs according to their biogenesis and size. EXOs are endosome-derived nanoscale vesicles, typically between 30 and 150 nm in diameter, with a lipid bilayer membrane whose lipid structure consists of phospholipids, cholesterol, ceramides and saturated fatty acid acyl chains [125]. In addition, certain surface markers, such as CD9, CD63, CD81, HSP70 and Tsg101, can be used to distinguish EXOs from other EVs [125]. EXOs are found in most body fluids, such as plasma, saliva, urine, pleural ascites, amniotic fluid, cerebrospinal fluid and breast milk [126]. They are rich in bioactive molecules, mainly proteins, lipids and nucleic acids (mRNA and microRNA), which can transfer signaling molecules between donor and recipient cells and regulate a variety of physiological and pathological processes [126, 127]. The double-layer membrane of EXOs not only provides protection but also maintains a controlled microenvironment that allows cargo to be transported over long distances [128]. Recent studies have shown that EXOs have attracted extensive attention in the field of regenerative medicine due to their excellent stability, biocompatibility, ability to penetrate the blood-brain barrier (BBB) [129], low immunogenicity, therapeutic targeting and high transport efficiency and have become one of the important emerging strategies for the treatment of PNI [130].

**Figure 4:**
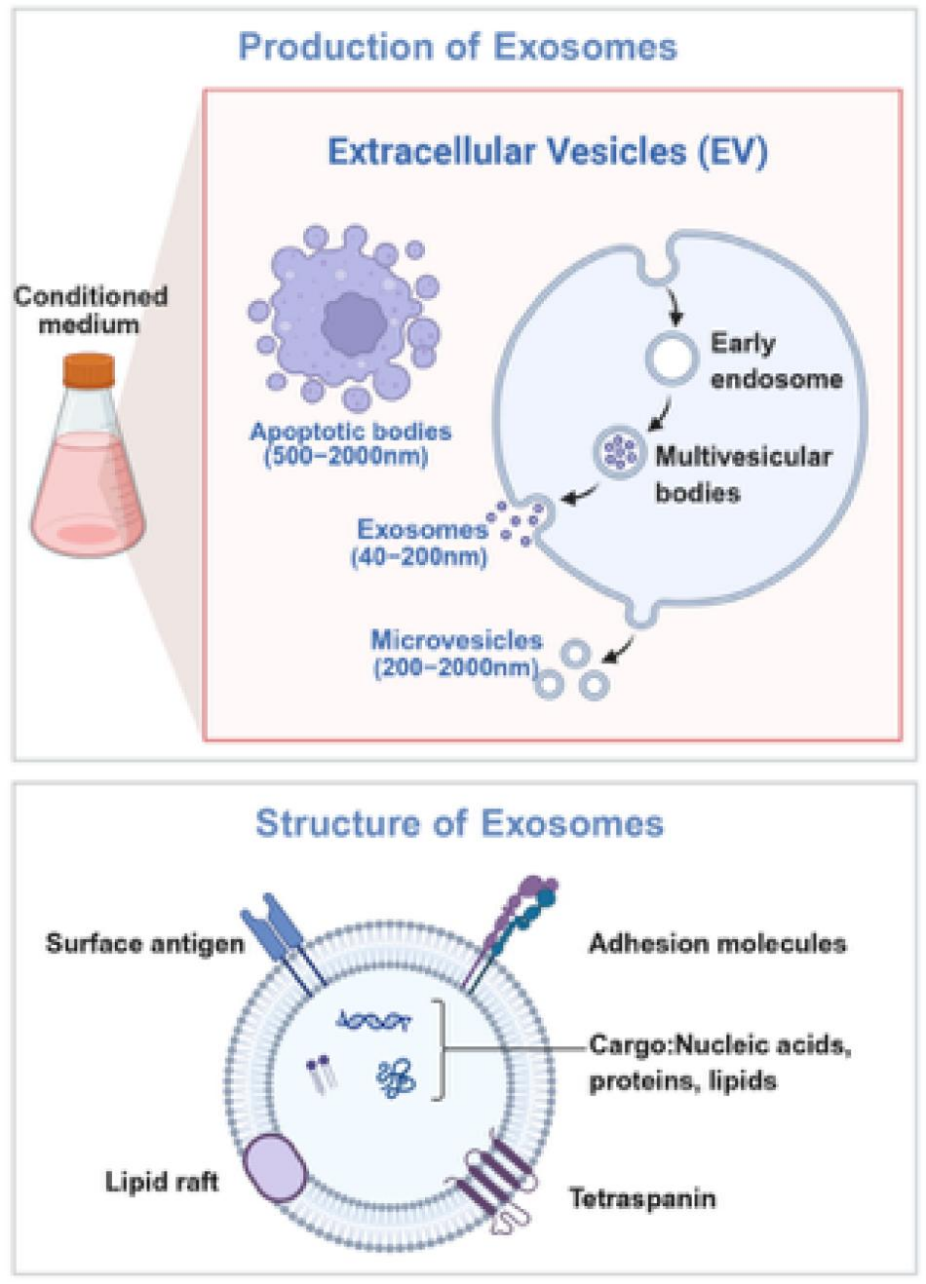
The schematic illustration of production and structure exosomes. EXOs are endosome-derived nanoscale vesicles, typically between 30 and 150 nm in diameter, with a lipid bilayer membrane whose lipid structure consists of phospholipids, cholesterol, ceramides and saturated fatty acid acyl chains. They are rich in bioactive molecules, mainly proteins, lipids and nucleic acids (mRNA and microRNA).

EXOs originate from the cell’s endosomal pathway (Figure 4). When the endosomal membrane invades to form multiple intracavitary vesicles (ILVs), these vesicles undergo a maturation process. At this stage of development, endosomes are called multivesicular bodies (MVBs). Vesicles within MVBs can undergo two different outcomes, in one case, they can fuse with lysosomes, causing their contents to be degraded; in the other case, they fuse with the plasma membrane, releasing ILVs as EXOs into the extracellular environment [131]. The biogenesis of EXOs involves two unique molecular processes, namely the endosome-dependent sorting transport complex (ESCRT) pathway and the ESCRT complex-independent pathway [132]. When EXOs are released into the extracellular environment, they reach their target tissues in three main ways. They can interact with lipid ligand receptors to activate target cells; the cell surface receptor is transferred to the recipient cell through the budding process; finally, the cell is internalized by fusion with the cell membrane, and the cytoplasmic contents of the donor cell are transferred to the recipient cell [133, 134].

In the research and application field of EXOs, the extraction and identification technology of EXOs constitutes the basis of research. So far, researchers have developed various methods to successfully isolate EXOs from different sources, such as ultrahigh-speed centrifugation, volume-based separation techniques, *in situ* polymer precipitation, immunoaffinity capture techniques and microfluidics [130, 135]. Currently, the gold standard method for isolating EXOs is ultracentrifugation, a simple and highly pure technique for obtaining EXOs based on differences in density and particle size, including differential ultracentrifugation and density layer ultracentrifugation [136]. However, this technique requires expensive ultracentrifuge equipment. Moreover, the centrifugation process may disrupt the structure of EXOs, resulting in their reduced biological activity [137]. The techniques for separating EXOs according to their size are ultrafiltration and size-exclusion chromatography, which do not require special large equipment and can handle larger volumes of samples, but EXOs may clog the ultrafiltration membrane, affecting the separation efficiency [138]. *In situ* polymer precipitation is a separation method based on changing the solubility of EXOs in solution, which is capable of processing a large number of samples and can complete the enrichment of EXOs in a relatively short time. However, the purity of EXOs obtained by this technique is relatively low, and the precipitation reagent may have a certain impact on the structure and biological activity of EXOs [139]. Another method for isolating EXOs is immunoaffinity capture technology, which is based on the interaction between antibodies and EXOs surface proteins [140]. The technology can selectively isolate EXOs of specific cell origin or with specific markers, and is able to maintain the biological activity of EXOs, but the need to purchase high-quality antibodies results in higher costs [141]. Microfluidic technology uses microstructures to finely adjust the physical characteristics (such as flow rate and pressure) and biochemical interactions (such as antigen–antibody binding and affinity) of fluids to achieve efficient separation, enrichment and analysis of EXOs [137, 142].

In recent years, with the in-depth study of EXOs, their potential application value has been continuously discovered. They can be used for tissue regeneration, drug delivery, gene therapy, vaccine development and as biomarkers for the diagnosis and prognosis of various diseases, such as cardiovascular disease, cancer, neurodegenerative diseases, skin regeneration, diabetes and for the improvement of immune function [125]. Exosomal proteins released by nerve cells, such as GAP43, neurogranin protein, SNAP25 and synaptic binding protein 1, have been shown to have potential applications in the diagnosis of Alzheimer’s disease and amnesia associated with mild cognitive impairment [143]. The levels of exosomal microRNAs such as miR-499, miR-194, miR-208 and miR-133 are associated with cardiovascular disease and are upregulated in patients with acute myocardial infarction and heart failure, suggesting potential diagnostic markers [144]. Li et al. [74] constructed a novel acellular tissue-engineered bone by combining EXOs derived from human ADSCs (hADSCs-EXOs) with polylactic acid-hydroxyacetic acid copolymer (PLGA) scaffolds, accelerating the repair of critical-sized skull defects in mice. In addition, a growing body of evidence also suggests that EXOs play an important role in promoting neural regeneration following damage to the PNS [145, 146]. Therefore, the potential of EXOs to treat PNI has become a topic of increasing concern, and they mainly play a role through the following aspects.

#### EXOs can stimulate Schwann cells activation

Schwann cells are glial cells in the PNS, originating from NCCs and arranged regularly in the peripheral nerves [147]. They establish myelin sheaths around the axons, a process that helps protect the structural integrity of the axons and enhances the speed of signal transmission [148]. After PNI, Schwann cells dedifferentiate, become activated and undergo phenotypic transformation [149]. Activated Schwann cells can proliferate, migrate and secrete a variety of neurotrophic factors, work with macrophages to clear nerve debris, create a favorable microenvironment for nerve regeneration and guide axon regeneration and re-myelination [150]. In recent years, studies have shown that EXOs can promote peripheral nerve regeneration by enhancing the biological activity of Schwann cells. Chen et al. showed that ADSCs-derived EXOs (ADSCs-EXOs) internalized by Schwann cells can promote the proliferation, migration, myelination and secretion of neurotrophic factors of Schwann cells, and then, improve the sciatic nerve regeneration of rats *in vivo*, which is mainly achieved by increasing the expression of corresponding genes [69]. Yin et al. confirmed that ADSCs-EXOs can reduce autophagy of Schwann cells induced by nerve damage by downregulating the expression of karyopherin subunit α 2 (Kpna2) by miRNA-26b, thereby enhancing myelin regeneration and promoting peripheral nerve repair [99]. In addition, MSCs-derived EXOs (MSCs-EXOs) can inhibit the apoptosis of Schwann cells and promote their proliferation by upregulating the expression level of anti-apoptotic gene Bcl-2 mRNA and downregulating the expression level of pro-apoptotic gene Bax mRNA [70]. This mechanism plays an important neuroprotective role after PNI. Haertinger et al. found that ADSCs-derived EVs (ADSCs-EVs) can significantly enhance the proliferation of Schwann cells in a time and dose-dependent manner. Their further analysis revealed that ADSCs-EVs enter Schwann cells through endocytosis, rather than binding or fusing with their plasma membrane. These EVs mainly accumulate around the nucleus and release the substances they carry through fusion with the endosomal membrane [151]. In addition, miRNAs in EVs primarily function by influencing the expression of genes in Schwann cells that respond to neural damage. The study also confirmed that these ADSCs-EVs contain mRNA for neurotrophic factors, further supporting their potential role in neuroprotection [151]. In addition to ADSCs-EXOs, it is believed that EXOs from other MSCs can also affect Schwann cells. Rao et al. found that EXOs derived from gingival mesenchymal stem cells (GMSCs) (GMSCs-EXOs) can significantly promote the proliferation of Schwann cells, and chitin catheters combined with EXOs were used *in vivo* to repair 10 mm peripheral nerve defects in rats [152]. Overall, these findings suggest that EXOs exert neuroprotective effects by influencing Schwann cells, providing a foundation for further enhancing the therapeutic efficacy of PNIs.

#### EXOs can promote the regeneration of axonal

EXOs can promote the regeneration of axons, which are important components of nerve cells and are responsible for transmitting nerve impulses. After nerve injury, the regeneration of axons is essential for restoring nerve signaling. To reconnect with the target cells, the axon must regenerate from the injury site and extend to the distal nerve [153]. In addition, axon regeneration can fill in the gaps in neural connections caused by injury, allowing broken or damaged neural networks to be restored to their integrity [154]. Interestingly, EXOs have been shown to have an impact on axon regeneration. Schwann cells can regulate nerve cell function and maintain normal nerve homeostasis. EXOs derived from Schwann cells (Schwann cells-EXOs) are important carriers of communication between Schwann cells and axons, which can regulate the regeneration process of axons [155]. Lopez-Leal et al. showed that the transformation of Schwann cells to repair phenotypes can be achieved by modifying the miRNA cargo of EXOs. For example, increased expression of miRNA-21 can promote axon regeneration by downregulating PTEN and PI3 kinase activation in the nervous system [156]. Lopez-Verrilli et al. found that dedifferentiated Schwann cells-EXOs are axon-specific internalized. These EXOs promote DRG nerve cell survival *in vitro* and increase axon regeneration, enhancing sciatic nerve regeneration after injury *in vivo* [157]. In addition, these EXOs also help to transform growth cone morphology into a pro-regenerative phenotype, thereby inhibiting the activity of GTPase RhoA, a protein involved in growth cone collapse and axon retraction [157]. Not only can Schwann cells-EXOs promote axon regeneration, but MSCs-EXOs also demonstrate the potential to promote axon regeneration. Zhao et al. have demonstrated that BMSCs-derived EXOs (BMSCs-EXOs) may significantly enhance neuron formation and promote axon growth through miRNA-mediated regulation of regeneration-related gene expression, such as VEGFA and S100b [158]. In addition, EXOs are rich in neurotrophic factors, such as BDNF, NGF and GDNF, which can effectively promote the survival, migration and growth of nerve cells after PNI. These factors promote the growth and differentiation of nerve cells by activating intracellular signaling pathways, enhancing the repair ability of the nervous system [159]. Bucan et al. showed that ADSCs-EXOs could stimulate the growth of DRG nerve cells by secreting neurotrophic factors *in vitro*, and could promote the regeneration of sciatic nerve after crush injury *in vivo* [160]. Moreover, GMSCs-EXOs have also been shown to enhance DRG axon regeneration [152]. In summary, these findings indicated that EXOs play a crucial role in axon regeneration and signal transmission, providing important support for peripheral nerve repair.

#### EXOs can alleviate inflammation

EXOs alleviate inflammation after nerve injury by regulating the local immune microenvironment. The inflammatory response provides critical support for neural repair, such as clearing obstacles, promoting regeneration and stabilizing the microenvironment, which is crucial to the neural repair process. In addition, although inflammation is the body’s natural response to injury, excessive inflammation may lead to secondary damage and affect nerve repair [161]. Therefore, targeting the inflammatory response triggered by peripheral nerve damage has become a therapeutic intervention. Numerous studies have shown that EXOs could effectively modulate the immune response after neural injury [162]. For example, mesenchymal stromal cells-derived EXOs could reduce neurovascular dysfunction caused by peripheral neuropathy in diabetic mice by reducing expression of pro-inflammatory genes, thereby improving functional recovery [163]. EXOs can also effectively regulate the release of inflammation-related cytokines. Ni et al. found that BMSCs-EXOs could reduce early neuroinflammation in mice with traumatic brain injury by modulating the polarization of microglia and macrophages, thus, exhibiting neuroprotective effects [164]. Ma et al. showed that in a sciatic nerve injury model, human umbilical cord mesenchymal stem cells (HUC-MSCs) derived EVs could aggregate into rat nerve defects, down-regulated pro-inflammatory cytokines IL-6 and IL-1β, up-regulated anti-inflammatory cytokines IL-10 and modulated inflammatory responses in injured nerves, thus, providing a favorable microenvironment for neural regeneration to improve functional recovery and promote neural regeneration [46]. Furthermore, EXOs could transform M1-type macrophages into M2-type macrophages by modulating the polarization state of macrophages, thereby promoting the repair process of injury. For example, Sun et al. showed that EXOs derived from HUC-MSCs (HUC-MSCs-EXOs) could prompt macrophages to switch from M1 to M2, which helps to suppress excessive inflammatory responses. *In vivo* findings indicated that these EXOs could improve functional recovery after neural injury by downregulating inflammatory cytokines such as TNF-α, MIP-1α, IL-6 and IFN-γ. These results suggested that HUC-MSCs-EXOs could promote the healing of nerve damage by reducing inflammation in damaged areas [47]. It has been reported that GMSCs-EXOs participate in the suppression of inflammation by modulating the secretion of IL-1 receptor antagonist (IL-1RA), thus, improving the efficacy of chitin ducts in the treatment of sciatic nerve injury in rats [152, 165]. In addition, there are two proteins in Schwann cells-EXOs, alphaB-crystallin and galectin-1, which can modulate the inflammatory response after nerve injury [166]. Overall, these findings suggested that the role of EXOs in immune regulation contributed to the effective regulation of neuroinflammation, which in turn created a microenvironment conducive to peripheral nerve regeneration.

#### EXOs can promote angiogenesis

EXOs play an important role in promoting vascular regeneration. Vascular regeneration is a key step in the peripheral nerve repair process, providing essential nutrients and oxygen for peripheral nerve repair. In addition to providing nutritional support, blood vessels can also act as a channel for the migration of Schwann cells, and vascular ECs can secrete bioactive molecules that promote neurite growth [167]. In recent years, studies have shown that bioactive molecules in EXOs can effectively promote the proliferation and migration of ECs and promote vascular regeneration. For example, Sun et al. found that hypoxic-treated Schwann cells-EXOs were able to enhance neural revascularization after PNI by increasing miR-21-5p expression [168]. Yang et al. demonstrated that ADSCs-EXOs could promote the migration and vascular regeneration of brain microvascular ECs after oxygen-glucose deprivation through the miR-181b-5p/TRPM7 axis, providing a novel therapeutic approach for the recovery from ischemic stroke [169]. In addition, Wang et al. demonstrated that ADSCs-EXOs induce macrophages to polarize towards M2 phenotype by activating the JAK/STAT6 signaling pathway. This process can promote the proliferation, migration and adhesion of M2 macrophages and inhibit the apoptosis of macrophages, thereby promoting angiogenesis and revascularization of ischemic lower limbs in diabetic mice [170]. According to reports, circulating endothelial progenitor cell-derived EXOs can enhance vascular remodeling and neurogenesis through the miR-126/PI3K signaling pathway, thereby reducing brain cell apoptosis and improving sensorimotor function [171]. In a word, these findings suggested that EXOs provide a new therapeutic strategy for the repair of PNI by promoting vascular regeneration.

With its unique biological advantages, EXOs therapy has shown broad application prospects in the field of regenerative medicine. Compared with traditional cell therapy, EXOs have low immunogenicity and are less susceptible to immune rejection. Their nanoscale size and good biocompatibility enable them to penetrate BBB and act directly on the central and PNS. EXOs have high stability and can be stored for a long time under low temperature conditions, which is convenient for transportation and storage. No cell expansion is required, avoiding the potential risk of tumor formation after cell transplantation. Despite this, EXOs therapy still faces some challenges. The efficiency of extracting and purifying EXOs is low, and the preparation process has not been fully standardized, which hinders their large-scale application in clinical practice. The biological activity of EXOs is influenced by the donor cell state and culture conditions, and there may be significant differences in function between EXOs from different sources. What’s more, the distribution, metabolism and clearance mechanisms of EXOs *in vivo* are not fully understood, which may affect their therapeutic effectiveness. The delivery efficiency and targeting of EXOs need to be further optimized, and more efficient delivery systems need to be developed to enhance their role in nerve injury repair [130,131, 138].

### Tissue engineering strategies

Cell therapy is an effective treatment strategy for PNI. However, this therapy also poses certain challenges, such as low survival rate of transplanted cells, insufficient regenerative capacity, uncontrolled differentiation, tumorigenesis, risk of capillary obstruction during infusion and low availability and low targeting of EXOs. To enhance the therapeutic potential of transplanted cells and EXOs, it is particularly important to combine them with biomaterials. This integration allows for the creation of an ideal microenvironment through tissue engineering to support cells. These structures not only provide the necessary three-dimensional (3D) structural support but also promote the targeted growth and functional recovery of cells, and promote the regeneration of damaged neural tissue, which is one of the important strategies in regenerative medicine (Table 2).

**Table 2:** Biomaterials in neural tissue engineering.

Types of biomaterials		Material source	Material characteristics	Mechanisms for promoting nerve repair	Advantages	Disadvantages	References
Natural biomaterials (polysaccharides)	Alginate	Extracted from brown algae and other algae plants.	Strong hydrophilicity, can form hydrogels and has good biocompatibility.	Load and slowly release nerve—active substances, creating a moist repair environment.	Can be rapidly formed by ionic cross—linking, facilitating operation.	Low mechanical strength and it is difficult to regulate its degradation process.	[172]
	Chitosan	The exoskeletons of crustaceans such as shrimps and crabs and the cell walls of fungi.	Has antibacterial properties, biodegradability and good film—forming properties.	Regulate the cell microenvironment and promote the binding and release of NGF.	Easy to process and form, and can be made into various nerve conduit forms.	Insufficient mechanical strength when used alone.	[55]
	Hyaluronic acid	Widely present in animal tissues, such as the comb, umbilical cord and vitreous body.	Highly hydrophilic and widely exists in the ECM.	Regulate cell proliferation, migration and differentiation and maintains the homeostasis of the cell microenvironment.	Strong moisturizing property, can promote cell—cell communication and has good biocompatibility.	Poor mechanical properties when used alone, fast metabolism in the body and cells do not adhere.	[38]
Natural biomaterials (proteins)	Gelatin	Prepared by partial hydrolysis of collagen in connective tissues such as animal skins, bones and tendons.	Good biocompatibility and biodegradability, rich in amino acids.	Provide cell recognition sites and promotes cell migration and differentiation.	Low cost, easy to obtain and process.	Poor mechanical properties and poor stability.	[173]
	Silk fibroin	Silkworm cocoons and spider silk.	Excellent biocompatibility and mechanical properties, and can be slowly degraded.	Support the attachment and growth of nerve cells and maintains cell viability.	Can be spun and formed into films, with diverse processing methods.	The long—term effects of degradation products are not yet fully understood.	[174]
	Collagen	Isolated from biological tissues such as bones, skin, tendons and blood vessels.	Good biocompatibility and biodegradability, and its structure is similar to the human ECM.	Provide a growth scaffold for nerve cells and promotes cell adhesion, proliferation and differentiation.	Wide source, low immunogenicity, good water—absorption capacity, and multiple separation methods are available.	Poor mechanical properties, and it is difficult to accurately control the degradation rate.	[175]
	Fibrin	Formed by blood coagulation factors in the human body during the blood coagulation process.	Naturally present in the human blood coagulation process and can form a three—dimensional network structure.	Simulate the ECM and promotes cell migration and axon growth.	Good hemostatic performance, can be formed *in situ*, and is beneficial for tissue repair.	Weak mechanical strength and easy to degrade rapidly.	[176]
	Keratin	Derived from tissues rich in keratin such as animal hairs, nails, feathers and hooves.	Derived from hairs and nails, with good mechanical properties and biocompatibility.	Provide attachment sites for nerve cells and promotes cell growth.	Renewable resource and can be processed into various forms.	The processing technology may affect its biological activity.	[177]
	Laminin	Mainly extracted from the basement membranes of animal tissues.	An important component of the ECM with cell recognition sites.	Promote the adhesion, survival and axon extension of nerve cells.	Specifically promotes nerve repair with high biological activity.	Difficult to extract and prepare and high cost.	[178]
Synthetic Biomaterials	Polylactic acid (PLA)	Synthesized by the polycondensation reaction of lactic acid monomers.	Adjustable mechanical properties and controllable degradation rate.	Can provide specific cell adhesion sites through surface modification.	Material properties can be customized according to requirements and the production process is mature.	Biocompatibility is slightly inferior to natural materials and degradation products may affect the local microenvironment.	[179]
	Polycaprolactone (PCL)	Prepared by the ring—opening polymerization of ε- caprolactone monomers under the action of an initiator.	Good flexibility and low melting point, facilitating processing.	Can be used as a carrier to continuously release nerve—active substances.	Can prepare nerve repair materials with complex structures and has a relatively low cost.	Long degradation time in the body.	[180]
	polyethylene glycol lactide (PGLA)	Synthesized by ring-opening copolymerization reaction of polyethylene glycol and lactide.	Biodegradability and certain mechanical properties, with good biocompatibility.	By adjusting the proportion, the degradation rate and surface properties can be controlled, which is beneficial for cell adhesion and the release of NGF loading.	Customize properties such as hydrophilicity and degradation rate according to requirements.	The synthesis process has relatively high requirements and a relatively high cost.	[181]
	Polyglycolic acid (PGA)	Prepared by the polycondensation of glycolic acid monomers or the ring—opening polymerization of glycolide.	High crystallinity and fast degradation rate.	Rapidly constructs a temporary scaffold to guide nerve regeneration.	High initial mechanical strength, suitable for short—term repair.	Too fast degradation rate may affect long—term repair effects.	[182]
	Polyurethane (PU)	Synthesized by the step—by—step polymerization reaction of polyols and polyisocyanates.	Good elasticity and mechanical properties and good chemical stability.	The surface properties can be designed to promote cell interactions.	Can withstand certain mechanical stress, suitable for dynamic tissue repair.	Biocompatibility needs to be further optimized and impurities may be introduced during the synthesis process.	[183]
	Polyhydroxyalkanoates (PHAs)	Synthesized by microbial fermentation using renewable carbon sources as substrates.	Good biocompatibility and biodegradability.	Support cell adhesion and proliferation and regulates cell behavior.	Can be naturally metabolized in the body, environmentally friendly.	High synthesis cost and difficult to process.	[184]
	Polyethylene glycol (PEG)	Prepared by the step—by—step addition polymerization of ethylene oxide with water or ethylene glycol.	Good water solubility, high biocompatibility and adjustable molecular weight.	Can modify the material surface to improve hydrophilicity and anti—protein adsorption and can be used as a drug carrier.	Can be used to prepare hydrogels and can effectively control drug release.	Lack cell recognition sites and needs chemical modification to better promote nerve repair.	[185]

Tissue engineering integrates the research results of cell biology and materials science to promote functional recovery after injury by simulating the physical and chemical environment of natural tissues [186]. Its core elements include cells, bioactive factors and scaffolds. Cells are one of the most critical elements in tissue engineering. During the repair of PNI, cells can directly promote the recovery of neural function by secreting growth factors and differentiating into different types of nerve cells [187]. Bioactive factors play a role in regulating cell function and promoting regeneration in tissue engineering. Biological factors, including neurotrophic factors, cytokines and growth factors, can regulate cell proliferation, differentiation and migration and promote nerve repair. For example, BDNF, neurotrophic factor can provide necessary growth support for nerve cells, promote axon regeneration and functional recovery [188]. The role of scaffolds in tissue engineering is to provide a supportive structure for cells, promote cell growth, differentiation and migration and simulate and restore the biomechanical properties of damaged tissues [189]. The ideal scaffold should have high biocompatibility, adjustable mechanical strength, high porosity, large surface area, the ability to provide physical support for connecting the proximal and distal stump of the nerve, the ability to mimic the physical and chemical properties of the ECM and the ability to replace damaged tissue with exogenous (transplanted) or endogenous cells with the correct tissue structure to achieve nerve regeneration [190]. The right chemical environment may be provided by biological materials that can carry cells or EXOs to provide nutrients and trophic factors to damaged areas. The biomaterials used to make scaffolds should have good biocompatibility, biodegradability, mechanical strength, cell adhesion and minimal immunogenicity, be nontoxic, non-teratogenic, noncarcinogenic and support nerve regeneration and recovery [18]. The selection of biomaterials directly affects the growth, migration and differentiation of cells, thus, affecting the effect of nerve repair. Biomaterials are mainly divided into natural-derived biomaterials, synthetic materials and composites [18].

Biomaterials from natural sources have good biocompatibility, biodegradability and the ability to support cell adhesion and proliferation, which makes them widely used in tissue engineering. Common natural biomaterials include collagen, chitosan, alginate, gelatin, silk fibroin and hyaluronic acid [191]. Collagen is a common structure that can be isolated from biological tissues such as bones, skin, tendons and blood vessels. As a highly flexible natural polymer protein, it is the main protein component of ECM. Due to its low immune response and high absorption rate in the body, it can be used to make artificial nerve guide conduits (NGCs) and deliver drugs [192, 193]. Guha Sarkar et al. developed a biodegradable *in situ* gel liposome gel (LP-Gel) system loaded with paclitaxel (PTX) for intrabladder drug delivery. Their research also showed that the LP-Gel platform system had the potential to develop into a promising intrabladder therapeutic platform with low side effects, extended drug retention time and excellent permeability [194]. Georgiou et al. designed type I collagen-based scaffolds loaded with Schwann cells, and *in vivo* experiments demonstrated that these scaffolds promoted axon regeneration in a 5 mm deficient rat model of sciatic nerve injury [195]. In addition, Chitose et al. successfully fabricated a targeted collagen scaffold containing cultured Schwann cells, which resulted in partial functional regeneration of a 20 mm long recurrent laryngeal nerve defect in a canine model [175]. Although collagen has shown significant clinical effects, it has low mechanical strength and poor maneuverability, and may not provide adequate structural support when treating large neural defects. These issues need to be addressed in future studies [196].

Recent studies have shown that chitosan, due to its good biocompatibility, can promote Schwann cells attachment and proliferation, support axon regeneration and reduce the formation of scar tissue, and has great potential in peripheral nerve regeneration [55]. For example, Wei et al. prepared chitosan nanoparticles coated with NGF, which *in vitro* experiments showed promoted the adhesion and proliferation of Schwann cells, and *in vivo* experiments demonstrated that they successfully repaired 10 mm nerve defects [197]. Another study found that chitosan nerve ducts inoculated with autologous bone marrow mononuclear cells promoted axon regeneration, which effectively repaired long-distance peripheral nerve defects [198]. Moreover, Bo et al. found that chitin scaffolds containing autologous nerve tissue can effectively promote sciatic nerve regeneration, enhance myelination and improve the recovery of nerve function [199].

Alginate is a common natural polysaccharide, usually extracted from brown seaweed, and its main components are manuronic acid and gururonic acid. Due to its strong hydration properties, good chemical flexibility, controllable biodegradability and good biocompatibility, it has become a common material in the treatment of nerve injury [56]. It has been reported that alginate is capable of cross-linking with divalent metal ions (especially Ca^2+^) to form hydrogels in solution. The hydrogel absorbs liquid to expand, forming a soft and elastic biomaterial that minimizes irritation to surrounding tissues and provides favorable conditions for nerve regeneration [200]. Wu et al. found that gelatin methacryloyl (GelMA) hydrogel can promote the growth of PC12 cells and axon growth, thereby promoting peripheral nerve regeneration [201]. In addition, Xu et al. developed an alginate scaffold with HUC-MSC-EXOs, which inhibits inflammation after nerve injury and alleviates nerve pain [202]. However, natural biomaterials require extensive purification processes and their chemical compositions are heterogeneous, which can lead to fluctuations in degradation rates and other mechanical properties [13].

Compared with natural biomaterials, synthetic materials offer greater flexibility in design and production, allowing them to adjust their mechanical properties and biodegradation rates [192]. For example, polylactic acid (PLA) [179], polyglycolic acid (PGA) [182, 203], polyethylene glycol lactide (PGLA) [204, 205], poly (ε-caprolactone) (PCL) [206] and polyurethane (PU) [207] had become the focus of neural repair research due to their good mechanical properties and adjustable degradation rates. For example, Reid et al. demonstrated *in vivo* that they repaired 10 mm of sciatic nerve damage in rats by fabricating neural catheters using PCL films [208]. However, the degradation products of synthetic polymers have acidic properties and poor biocompatibility, which may trigger an inflammatory response to surrounding tissues, thus, hindering the repair and regeneration of peripheral nerves. To overcome the inherent limitations of individual biomaterials, composites combining natural and synthetic polymers have received extensive attention [209]. For example, Song et al. prepared salidroside/collagen/polycaprolactone (PCL) neural catheters, which were shown to repair sciatic nerve defects in rats *in vivo* [210]. Researchers prepared laminin and PCL blend nanofibers to simulate the peripheral nerve basement membrane. Aligned PCL nanofibers significantly improved motor function; aligned laminin blend nanofibers yielded the best sensory function recovery [211]. Using electrospinning, nanofibrous neat PCL and PCL/multiwalled carbon nanotubes composite scaffolds were prepared in random and aligned morphology. The inclusion of multiwalled carbon nanotubes reduced the mechanical strength of nanocomposite scaffolds compared to neat PCL [212]. Researchers have designed a dual-functional scaffold that combines the neuroprotective and antioxidant capabilities of Bacopa monnieri extract with the mechanical and electrical characteristics of PCL-collagen fibers reinforced with multi-walled carbon nanotubes.

This biofortified scaffold, designed for nerve tissue engineering, takes advantage of the synergistic effects of electrical conductivity and plant-based bioactivity to facilitate peripheral nerve regeneration [213]. Nagarajan et al. developed a nanofibrous scaffold using PCL as a foundation loaded with antioxidant graphene oxide and coated this bioscaffold with Schwann cell acellular matrix. *In vitro* studies revealed both antioxidant and remyelination properties of the developed bioscaffold [214]. Gu et al. found that chitosan-PGLA nerve grafts combined with bone marrow mononuclear cells could effectively repair 50 mm median nerve defect and 80 mm ulnar nerve defect [215]. In addition, tubular prostheses containing PCL and collagen can also be utilized to enhance peripheral nerve regeneration [180]. The composite scaffold has good mechanical properties and cellular affinity, which holds great promise for promoting the progress of nerve regeneration.

Although biomaterials and scaffolds have significant advantages in the treatment of nerve injuries, they still face many challenges. The selection of biomaterials and the design of scaffolds need to comprehensively consider factors such as biocompatibility, degradability, mechanical properties and cell compatibility, and the regulation and optimization of these factors still need to be further studied. Existing scaffolds often fail to optimize the microenvironment required for cell growth while providing structural support. In addition, the functional design of scaffolds also faces multiple challenges such as how to accurately transmit cell growth signals, promote nerve cell growth and restore nerve function.

In recent years, researchers have tried to precisely design the structure of the scaffold through 3D printing technology to better adapt to different types of nerve damage [216, 217]. Three-dimensional printing technology, also known as additive manufacturing, is a type of rapid prototyping technology that creates 3D objects by stacking materials layer by layer. Compared with traditional subtractive manufacturing methods, 3D printing technology can precisely control the structure and shape of objects, and can be directly customized according to computer models [218]. In the field of nerve damage repair, the advantages of 3D printing technology are particularly apparent. Traditional neural repair methods typically rely on obtaining donor materials from outside or fabricating scaffolds from synthetic materials, but these methods often fail to fully simulate the complex structure of neural tissue and are difficult to meet the individual needs of patients. However, 3D printing technology can accurately create a scaffold that exactly matches the shape and size of the injury site based on the patient’s anatomical data, avoiding the limitations of traditional methods. Through digital design, 3D printing can precisely adjust the structure, size, porosity and other aspects of the scaffold, providing an ideal microenvironment for cell growth [219]. The researchers predicted the shape deformation of spatially patterned hydrogels with defined filling angles by calculating various 3D printing structures, and then, carried out experimental verification. The results showed that 3D printed hydrogel with preprogrammed filling patterns could rapidly self-roll into a tube *in vivo*, which could be used as a nerve guiding tube for repairing sciatic nerve defects [220]. Researches have shown that 3D-printed scaffolds can promote cell attachment and growth, and help form neural tissue-like structures [221]. For example, Gu et al. developed a printable bioink that is capable of rapidly gelatinizing through stable cross-linking to form porous 3D scaffolds that encapsulate human NSCs capable of differentiating and replacing lost function and/or supporting the growth of other functional nerve cells and glial cells [222]. A bioink composed of polyvinyl alcohol and cerium oxide nanoparticles, developed using a dual crosslinking method of citric acid and sodium hydroxide has achieved good printability of the bioink and shape fidelity of the bioprinting structure. This multimodal bioink can serve as both a cell carrier and a free radical scavenger for the treatment of peripheral nerve damage [223]. Bociaga et al. have shown that bioprinting technology can create scaffolds with excellent microstructures that promote cell growth [224]. Moreover, 3D printing technology also holds promise for printing tissue components, such as grafts and organs [225]. The study showed that the use of microsphere-loaded bioinks allows the printing of scaffolds with NPCs derived from human induced iPSCs (hiPSCs), which could promote neural tissue repair [176]. Another study evaluated the neural regeneration capacity of NGCs fabricated by 3D printing and multifunctioned using canine ADMSCs with heterofibrin biopolymers (HFB) as cell scaffolds. Experimental results show that printing scaffolds can promote sciatic nerve regeneration after injury [226].

In addition to simple 3D printing of catheters and loaded cells, 3D printing technology is also used to integrate growth factors and EXOs into neural conduits. Litowczenko et al developed GrooveNeuroTube is composed of hyaluronic acid methacrylate and gelatin methacrylate hydrogel, incorporating active agents (growth factors and antibacterial agents) encapsulated within an NGC conduit made of 3D-printed PCL grid fibers. Research shows that GrooveNeuroTube significantly promoted migration of dorsal root ganglion neuronal cells and can effectively support sustained neural cell migration and maturation over extended periods [227]. A 3D printing process of NGCs using PCL and GelMA integrated with a thermostable fibroblast growth factor 2. At 12 weeks, following a long-gap nerve injury in rats, NGC implantation enhanced sensory and motor recovery, improved electrophysiological function and promoted morphological and ultrastructural nerve reorganization and regeneration [228]. Fabricate an aligned topological scaffold by combining the 3D printing and electrospinning to exert synergistic topographical cue for peripheral nerve regeneration and modify the internal microenvironment by filling the lumen with umbilical cord-derived decellularized ECM hydrogels and EXOs. Herein, the NGCs provided obvious guidance and proliferation to schwann cells and PC12 *in vitro* due to the sustained-release effect of decellularized ECM hydrogels and the outstanding proliferation-promoting role of EXOs. The NGCs were surgically implanted *in vivo* to bridge a 12-mm gap sciatic nerve defect in rats and it had a satisfactory effect in reestablishment of the sciatic nerve, including the recovery of motor functions and the myelination [229].

Although 3D-printed neural scaffolds have shown great potential in experimental studies, their clinical application still faces many challenges. First of all, the matching between the degradation rate of the scaffold and the nerve regeneration rate needs to be further optimized. Too fast degradation may lead to the loss of support of the scaffold, and too slow degradation may affect tissue integration. Second, the stability and cell viability of bioprinting technology still need to be further improved to ensure that nerve cells remain active during printing and culture. In addition, the immune rejection, safety assessment and long-term efficacy studies involved in the clinical transformation process still need to be further explored.

### Gene therapy strategies

As an important part of regenerative medicine, gene therapy aims to introduce unnatural and therapeutic genes into living cells to promote the repair of tissues or organs. The application of gene therapy in peripheral nerves aims to promote the regeneration of damaged nerve cells, or to create a favorable microenvironment at the distal nerve stump through Schwann cells to promote the regeneration of axons [230]. Gene therapy applications in neural regeneration include gene delivery to the dorsal root ganglion (DRG) and Schwann cells. Viral vectors are the most effective way to introduce genes into cells. A viral vector is a modified virus that has lost its ability to replicate, but has the ability to attach and enter cells, while delivering genes to the nucleus [230].

Adeno-associated virus (AAV) vectors are widely used in human applications due to their low immunogenicity, lack of endogenous genome and high gene transfer capacity [231]. For example, Andrews et al. delivered alpha 9-integrin, a transmembrane receptor that mediates cell adhesion, proliferation and migration, into peripheral nerves via AAV vectors. Their experiments showed that forced expression of α9 integrin can enhance the axon growth of DRG nerve cells *in vitro* and *in vivo* [232]. Subsequent studies found that AAV vector-mediated Kindlin expression, an intracellular activator of integrin signaling, promoted the regeneration of the central axons of damaged DRG nerve cells *in vivo* [233]. In addition, the AAV vector was able to inhibit the activity of mammalian sterile 20-like kinase 3b (Mst3b) by delivering small hairpin RNA into mouse DRG, thereby inhibiting axon growth [234]. AAV vector-mediated bone morphogenetic factor 4 (BMP4) expression promotes axon regeneration by reactivating the transcription factor Smad1 signaling pathway [235].

In recent years, gene therapies have also targeted Schwann cells, with the aim of creating a microenvironment conducive to neural regeneration. Viral vector-mediated gene delivery to Schwann cells in peripheral nerves can be used to maintain denervated nerves in a state that sustainably supports nerve fiber regeneration, thus, ensuring that axons can grow to distal limbs and reconnect skin and muscle cells [236]. For example, Eggers et al. showed that lentiviral vector-mediated glial cell line-derived neurotrophic factor (GDNF) is overexpressed in the ventral horn, and that GDNF can spread from the transduced abdominal root to the ventral spinal cord, thereby enhancing axon growth [237]. In addition, the VEGF gene can induce angiogenesis. In a rat sciatic nerve injury model, VEGF was injected into the local environment, and it could play a positive role in nerve regeneration, myelination and reinnervation of target organs in a dose-dependent manner [238].

Although gene therapy has shown significant potential in laboratory studies, its application to clinical treatment still faces challenges. First, the efficiency and safety of gene delivery remain an urgent issue to be addressed. For example, the application of viral vectors may provoke an immune response. Second, the complexity of neural regeneration and individual differences are also important challenges that limit its clinical application. The repair process after nerve injury is influenced by many factors, including the degree of damage, the timing of treatment and the effectiveness of gene introduction. Furthermore, the clinical application of gene therapy urgently needs to address ethical issues, especially with regard to genetic modification, which may involve concerns about genetic privacy, long-term effects and other aspects [239]. Therefore, to ensure the effectiveness and safety of clinical treatment, more in-depth studies and multicenter clinical trials are needed to provide safer and more effective treatment strategies for patients with PNI.

## Conclusions and prospects

PNI is a common clinical disease, which significantly affects the quality of life of patients [240–242]. PNI can lead to loss of nerve function, rupture of blood vessels and disruption of nerve cell interactions with surrounding tissue. The repair effect after PNI is usually poor. Studies showed that only about 3% of patients regain sensory function, while less than 25% regain motor ability [243, 244]. Although autologous nerve transplantation is the gold standard treatment for long-segment nerve defects, it can lead to secondary surgical injuries such as motor dysfunction, pain and scarring problems, nerve reinjury and vascular damage [187]. What’s more, while significant progress has been made in the pathophysiology of nerve damage and regeneration, peripheral nerve repair remains a major clinical challenge. Therefore, it is necessary to explore new strategies to create an ideal regenerative microenvironment and further enhance the therapeutic effect on PNI.

**Figure 5:**
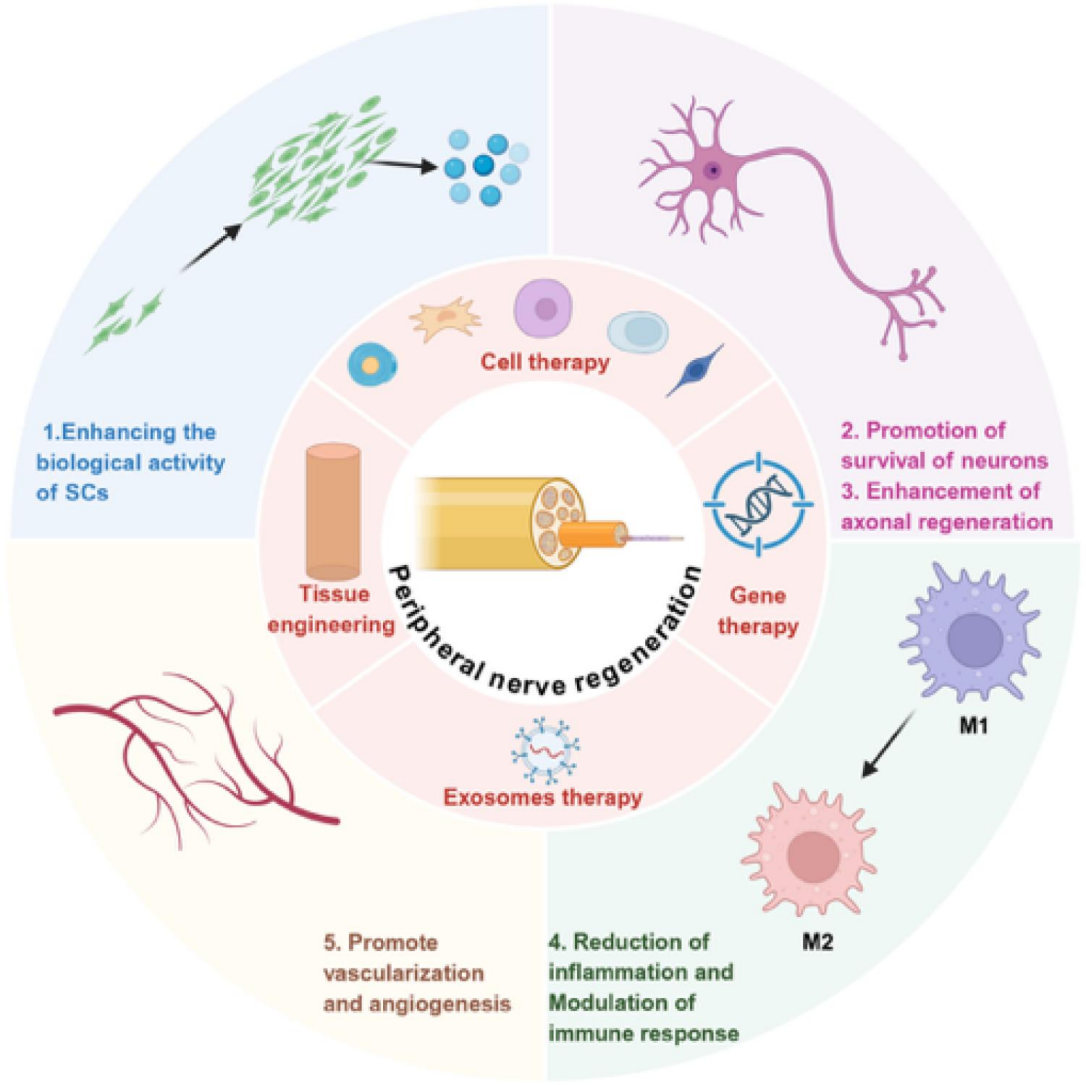
Schematic diagram of the function of regenerative medicine in peripheral nerve regeneration. Regenerative medicine strategies through enhances Schwann cell biological activity, promotes neuron survival, accelerates axonal regeneration, modulates macrophage polarization to reduce inflammation, promotes angiogenesis, provides structural support via biomaterial scaffolds and regulates the immune microenvironment and targets specific molecular pathways to enhance peripheral nerve regeneration. These strategies synergistically address key pathological barriers to improve functional recovery.

In recent years, regenerative medicine has made significant progress in the treatment of peripheral nerve injuries (Figure 5). From cell therapy and stem cell technology to tissue engineering, 3D printing scaffold technology [245–247] and gene therapy, an increasing number of novel therapeutic strategies have been proposed and shown promising results.

The potential of stem cell therapies, particularly MSCs and iPSCs, in repairing neural damage has been widely recognized [248, 249]. These cells can differentiate into nerve cells and secrete a variety of neurotrophic factors, promoting nerve regeneration and repair and greatly improving functional recovery after nerve injury [250]. In addition, stem cells can also enhance the effectiveness of neural repair by reducing inflammation and activating endogenous neural repair mechanisms [251]. However, despite the promising laboratory results of cell therapies, overcoming the problems of cell survival, migration and integration with host tissues in clinical applications remains an urgent challenge.

Recently, paracrine factors of cells have become increasingly popular, especially Exos, which have been shown to play a role in promoting PNI repair [245, 252]. Although the favorable results of Exos therapy in preclinical settings are encouraging, as an unprecedented treatment approach, there are still some issues that need to be addressed before applying it to clinical settings. First, the lack of GMP compliant large-scale production technology hinders the transition of Exos therapy from preclinical research to clinical research. From 3D cell culture to bioreactors, various methods for reproducing cell sources have been applied, but they require further improvement to effectively meet the clinical dose requirements of Exos. Second, in the absence of effective separation techniques, EVs precipitate together with other unwanted molecular pollutants, which hinders the clinical translation of therapeutic agents [253]. Third, the inability to accurately characterize and quantify the cargo of EVs, as well as the inability to target them to specific receptors, has raised concerns about the targets. Therefore, to guarantee safe and effective therapeutic outcomes, a series of issues spanning the manufacture, accurate characterization and quantification of EVs must be properly resolved to preclude potential side effects.

Biological scaffolds can provide physical support and further enhance the effectiveness of nerve repair through surface modification or combination with cells and neurotrophic factors [254, 255]. Although biological scaffolds demonstrate great neural repair potential, their long-term effectiveness and rate of degradation remain questionable. Some scaffold materials degrade too quickly to provide adequate support, and some scaffold materials may cause local inflammation [256, 257]. Therefore, developing biomaterial scaffolds with better performance is an important direction for future research. Specifically, the key priorities of such research include optimizing the degradation rate of tissue engineering scaffolds, reducing material induced local inflammation and developing clinically applicable scaffolds that balance structural support and biocompatibility.

Gene therapy has also made some preclinical progress, improving the effectiveness of nerve repair by delivering specific genes directly to the damaged site, and has a more durable and precise effect than traditional drug delivery [258]. However, the safety and long-term effectiveness of gene therapy also require further verification. One of the limitations currently hindering clinical translation is the method of transmitting genes to the nervous system. To this end, the latest advances in biomedical engineering provide a choice for the use of bioactive molecules and hydrogels to deliver genes to the neural microenvironment continuously and controllably. In addition, the process of nerve regeneration is usually slow, and in the case of extensive nerve damage, the recovery rate is often difficult to meet the needs of functional recovery. During nerve regeneration, scar tissue may form, restricting the growth of axons and leading to incomplete functional recovery. The long-term effects and complications of treatment, such as immune rejection, ectopic regeneration or tumor formation, remain major challenges facing regenerative medicine today [60].

In recent years, combination therapy has become a research hotspot in the field of regenerative medicine. By combining stem cell therapy, biological scaffolds and gene therapy, the repair efficiency of peripheral nerve damage has been successfully improved. For example, the use of stem cells in combination with biological scaffolds can enhance the survival and function of stem cells and promote the local release of NGF [259, 260]. Moreover, the use of gene therapy in combination with stem cells can further enhance the neural regeneration capacity of stem cells [261, 262]. Therefore, in the future, comprehensive therapeutic strategies combining gene therapy, molecular biology and cell engineering are expected to provide more effective solutions for the repair of peripheral nerve injuries.
